# The Role of STAT-3 and IL-26 Signaling Pathways in Leiomyoma Pathophysiology

**DOI:** 10.3390/jcm14176021

**Published:** 2025-08-26

**Authors:** Senol Senturk, Mehmet Kagitci, Tolga Mercantepe, Recep Bedir, Nalan Kuruca

**Affiliations:** 1 Department of Gynecology and Obstetrics, Faculty of Medicine, Recep Tayyip Erdogan University, 53100 Rize, Turkey; mehmet.kagitci@erdogan.edu.tr (M.K.); nalan.kuruca@erdogan.edu.tr (N.K.); 2 Department of Histology and Embryology, Faculty of Medicine, Recep Tayyip Erdogan University, 53100 Rize, Turkey; tolga.mercantepe@erdogan.edu.tr; 3 Department of Pathology, Faculty of Medicine, Recep Tayyip Erdogan University, 53100 Rize, Turkey; recep.bedir@erdogan.edu.tr

**Keywords:** leiomyoma, uterus, STAT-3, interleukin-26

## Abstract

**Background**: Uterine leiomyomas are the most common pelvic tumors in women of reproductive age. There is no clear conclusion in the literature regarding the pathophysiology of these conditions. STAT proteins stimulate the transcription of target genes. STAT-3 leads to an increase in VEGF levels and plays a role in tumorigenesis. IL-26 and other cytokines are vital immune response mediators. Cytokine dysregulation affects the immune response of various organs and tissues, making them prone to various diseases, such as inflammation, infection, and tumors. **Methods**: In the present study, we aimed to determine whether STAT-3 and IL-26 play a role in the development of uterine leiomyoma. This case–control study included 38 patients who underwent hysterectomy due to uterine leiomyoma and 30 patients who underwent hysterectomy due to non-organic benign gynecological causes other than myoma. Sections from the myometrium of the control group and the leiomyoma tissue of the case group were subjected to immunohistochemical staining for STAT-3 and IL-26. **Results**: When the uterine tissue sections of the control group incubated with STAT-3 were examined under a light microscope, the smooth muscle and fibroblast cells in the myometrium were STAT-3-negative, while the number of smooth muscle and fibroblast cells showing strong STAT-3-staining in the leiomyoma sections was high. When the uterine tissue sections incubated with IL-26 were examined under a light microscope, the normal smooth muscle and fibroblast cells in the control group were IL-26-negative, while there was an increase in the number of cells showing strong IL-26-staining in the leiomyoma smooth muscle and fibroblast cells. **Conclusions**: Our findings show that STAT-3 and IL-26 levels are significantly increased in uterine leiomyomas, and this increase may play a role in the growth and progression of uterine fibroids. The current results may enable the development of innovative treatment options, as they demonstrate the role of novel pathways in the formation of uterine fibroids.

## 1. Introduction

Uterine leiomyomas (fibroids or myomas) are the most common pelvic tumors in women of reproductive age. They usually cause menstrual irregularities, chronic pelvic pain, and infertility [1]. They occur in at least half of American women of reproductive age, and their frequency and size increase with age [2]. Fibroids represent an enormous public health burden for women and an economic cost to society. Various medical and surgical modalities are used to manage these patients. Although studies continue to be conducted on the best surgical modalities, the literature is not sufficiently clear on the pathophysiology of uterine fibroids. Strategies are needed to prevent, limit, and non-surgically treat the growth of these tumors.

Signal transducer and activator of transcription (STAT) proteins were identified in the early 1990s in conjunction with interferon (IFN)-mediated regulation of gene transcription [3]. Today, it is known that various cytokines induce different STAT proteins. Seven STAT proteins have been identified in mammalian cells. These are known as STAT-1, STAT-2, STAT-3, STAT-4, STAT-5a, STAT-5b, and STAT-6. The stages of cytokine-mediated STAT protein activation are as follows: cytokines bind to their cognate receptors on the cell surface. Subsequently, oligomerization occurs. This oligomerization stimulates Janus kinase (JAK) proteins associated with the receptor via cross-phosphorylation. Activated JAKs bind to type I and II cytokine receptors and phosphorylate tyrosine residues on these receptors and on other JAKs. This phosphorylation allows STAT proteins to dimerize, forming homodimers or heterodimers, and translocate to the cell nucleus. Once in the nucleus, STAT proteins interact with specific response element sequences on DNA, stimulating the transcription of target genes. Uncontrolled STAT-3 and STAT-5 activities play a role in malignant transformation. STAT proteins are involved in carcinogenesis through two mechanisms, one of which is the continuous activation of STAT proteins. STAT-3 activity leads to an increase in vascular endothelial growth factor (VEGF) levels and plays a role in tumor angiogenesis [4,5].

The upregulation or downregulation of cytokines, which are vital mediators of the immune system, influences the development of diseases such as inflammation, infection, and cancer. IL-26, a member of the IL-10 family and IL-20 subfamily, exhibits diverse effects in various diseases, owing to its unique cationic structure. IL-26 is secreted by Th17 cells and is considered a semi-qunatiproinflammatory cytokine. By binding to the IL-26 receptor complex (IL-10R1/IL-20R2), it induces multiple signaling mediators, particularly STAT-1/STAT-3 [6]. Due to its role in many pathological conditions, including inflammation, assessing IL-26 expression in tissues and biological fluids can be valuable, particularly for disease monitoring and prognosis [7,8,9,10]. Therefore, analyzing IL-26 expression in the myometrium and fibroids may be important for determining the role of cytokines in the development of leiomyomas. This study aimed to immunohistochemically detect STAT-3 and IL-26 expression in leiomyoma tissues and determine whether these signaling pathways play a role in leiomyoma pathophysiology.

## 2. Materials and Methods

### 2.1. Location of the Study

RTEU Training and Research Hospital.

### 2.2. Type of Research, Population, Sample, and Research Group

This case–control retrospective study included 38 patients aged 35–55 years who underwent hysterectomy due to uterine myoma at the RTEU Education and Research Hospital over a period of 5 years as the study group and patients who underwent hysterectomy for benign reasons other than fibroids as the control group. After extracting the records of patients who had undergone hysterectomy within the last five years from our archives, the final pathology reports of the surgical specimens were obtained from the pathology department. The indications for hysterectomy and the results of the pathology reports were recorded for each patient. Patients with pathological reports indicating uterine leiomyomas were included in the study group. The indication for hysterectomy in the study group was the presence of uterine fibroids. The control group comprised patients who underwent hysterectomy for benign gynecological reasons other than fibroids.

Tissue samples taken from some of the removed material (fibroid material of the study group and normal myometrium tissue of the control group) in the two groups of patients who underwent hysterectomy for their indications were evaluated immunohistochemically for STAT-3 and IL-26 activity. Since the primary outcome variable in the study was the staining of histopathological samples (such as hematoxylin and eosin) and these samples were used more than once, a sample size calculation was not required. Indications for hysterectomy in the myoma group were single or multiple uterine fibroids of various locations. In the control group, 14 patients underwent hysterectomy for dysfunctional uterine bleeding, 6 for multiple endometrial polyps, and 10 for grade 3 uterine prolapse. Endometrial polyp patients were those who had undergone hysteroscopic polypectomy but whose polyps had recurred.

Considering the roles of IL-26 and STAT-3 in tumorigenesis, angiogenesis, and inflammation [4,5,6,7], patients who were likely to affect the expression of these two cytokines were excluded from the study: those taking antiandrogen and lipid-lowering drugs with a history of intrauterine device insertion, Asherman syndrome, pelvic inflammatory disease, preoperative infectious disease, endometriosis, hydrosalpinx, previous pelvic surgery, recurrent abortion, and systemic and/or rheumatologic disease causing inflammation.

### 2.3. Ethical Approval

This study was conducted in compliance with the principles of the Declaration of Helsinki and was approved by the Ethics Committee of Recep Tayyip Erdoğan University (approval number: 2022/221). Written informed consent for the hysterectomy was obtained from all participants in the leiomyoma and control groups. Owing to the retrospective nature of the study, ethics committee approval was deemed sufficient for resection of paraffin blocks and immunostaining.

### 2.4. Histopathological Analysis

Biopsy specimens of uterine tissues were placed in tissue-tracking cassettes (Isolab GmbH, Eschau, Germany) and fixed in 10% phosphate-buffered formalin (Sigma-Aldrich, Darmstadt, Germany) for 24 h. Following fixation, dehydration (with increasing ethanol series, Merck KGAa, Darmstadt, Germany), mordanting (xylol, Merck KGAa, Darmstadt, Germany), and embedding in paraffin (Merck KGAa, Darmstadt, Germany) were performed according to routine histological follow-up procedures using a tissue-tracking device (Thermo Scientific Shendon Citadel 2000, Cheshire, England). Next, uterine tissue samples removed from the tissue-tracking cassettes were embedded in hard paraffin using a tissue embedding device (Leica 1150EGÜ, Leica Biosystems, Wetzlar, Germany) and blocked in tissue embedding cassettes (Merck KGAa, Darmstadt, Germany). The tissue samples were sectioned at 4–5 µm thickness with a rotary microtome (Leica RM2525, Lecia Biosystyems, Wetzlar, Germany) and stained with Harris hematoxylin and Eosin G (H&E; Merck, GmbH, Darmstadt, Germany).

### 2.5. Immunohistochemical Analysis

A total of 2–3 micrometer thick sections of myoma or myometrium tissue were placed on glass slides. The sections were stained with STAT-3 (ab109085, Abcam, Cambridge, UK) and IL-26 (ab224198, Abcam, Cambridge, UK) antibodies using a Bond-Max model (Leica Biosystems, Wetzlar, Germany) for automated immunohistochemical staining and in situ hybridization. For this purpose, uterine biopsy tissue sections were deparaffinized using Bond Dewax solution. Dehydrated peroxidase blockade was performed on biopsy tissues. Antigen retrieval was performed by heating the samples in ER2 solution (Leica Biosystems, Wetzlar, Germany) for 20 min. The cells were incubated with STAT-3 and IL-26 antibodies for 60 min. Tissues treated with secondary antibodies (ab205718, Abcam, Cambridge, UK) were stained with diaminobenzidine (DAB) using the Bond Polymer Refine Detection kit (Leica), and sections of biopsy specimens were stained with Harris hematoxylin (Bond Polymer Refine Detection, Leica, Wetzlar, Germany) for 10 min. After staining, the uterine tissue biopsy sections were covered with Entellan (Merck Gmbh, Darmstadt, Germany), examined, and photographed using a microscope. To assess nonspecific staining, negative controls were obtained by applying an equivalent amount of mouse or rabbit-specific IgG, instead of the primary antibody, to a portion of the uterine tissue samples.

Detailed information on the trichrome staining method can be found in the literature [11]. Briefly, paraffin blocks containing myometrium and fibroid tissue were sectioned into 5 μm sections, heated, and mounted on a slide. Following deparaffinization with xylene, the sections were passed through a graded series of alcohol and rehydrated. The sections were treated with Weigert iron hematoxylin, distilled water, Biebrich red fuchsin, phosphotungstic-phosphomolybdic acid, aniline blue, and glacial acetic acid [11]. The intensity of immunoreactivity in uterine biopsy sections stained with STAT-3 and IL-26 was determined using semi-quantitative analysis (Table 1).

To avoid errors in the assessment of immunopositivity in our study, we included a negative isotype control. As the isotype control, we used the TRKB primary antibody (ab18987, Abcam, UK), which has no affinity for endometrial tissue.

### 2.6. Semi-Quantitative Analysis

Tissue samples incubated with primary antibodies against STAT-3 and IL-26 were scored as shown in Table 1. Each preparation was scored by two histopathologists under a light microscope using 20 randomly selected fields at different magnifications (×20 and ×40). The histopathologists were blinded to the experimental group assignments. The immunostaining intensities of leiomyoma and myometrium samples were determined using the histo-score formula (histo-score = width × intensity). The extension h-score = (1 × % weak staining) + (2 × % moderate staining) + (3 × % strong staining) values were taken as the basis, while for density, (0: none, +0.5: little, +1: low, +2: moderate, +3: severe) values were taken [12].

### 2.7. Statistical Analysis

The data obtained as a result of semi-quantitative analyses were analyzed using the Shapiro–Wilk test, Q-Q plots, Kurtosis-Skewness values, and Levene’s tests using SPSS 20.0 (IBM Corporation, Armonk, NJ, USA) statistical software, and the conformity to normal distribution was evaluated. Graphs were generated using GraphPad Prism 10.4.2 software (GraphPad Software, San Diego, CA, USA). Data are presented as mean ± standard error of the mean (SEM) for continuous variables. Statistical differences between groups were analyzed using an independent sample *t*-test. Statistical significance was set at *p* < 0.05.

## 3. Results

The indications for hysterectomy in the myoma group were single or multiple uterine fibroids at various locations. In the control group, 14, 6, and 10 patients underwent hysterectomy for dysfunctional uterine bleeding, multiple endometrial polyps, and grade 3 uterine prolapse, respectively. Patients with endometrial polyps underwent hysteroscopic polypectomy but experienced a recurrence of polyps.

### 3.1. Histopathologic Findings

**Figure 1:**
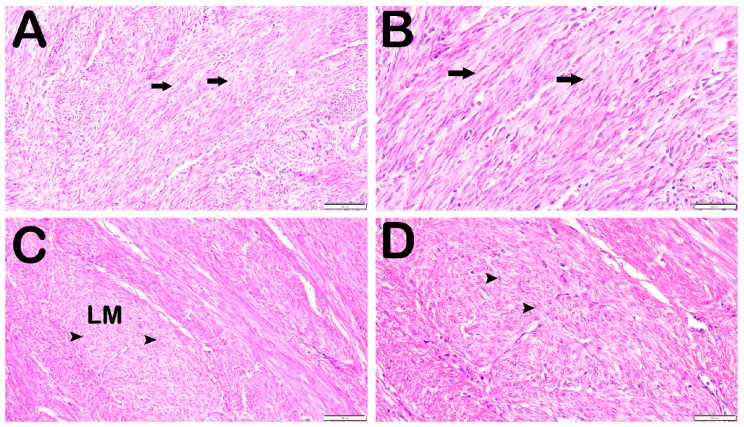
Representative light microscopic view of the uterine tissue sections (H&E staining). (**A**) (×20)–(**B**) (×40) Control Group: myometrium sections showing normal smooth muscle cells (arrow). Case Group (**C**) (×20)–(**D**) (×40): sections of leiomyoma samples showing dense cellular content with diffuse collagen units (arrowheads).

**Figure 2:**
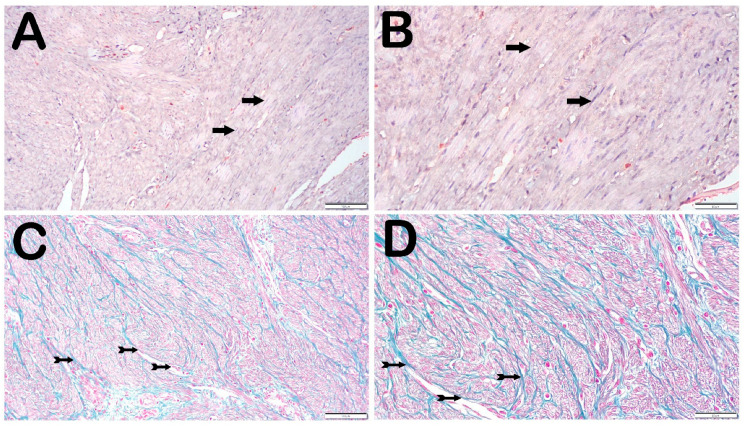
Representative light microscopic view of sections of biopsy specimens of the uterine tissue (Masson’s trichrome stain). (**A**) (×20)–(**B**) (×40) Control group: myometrium sections showing normal smooth muscle cells (arrow). (**C**) (×20)–(**D**) (×40) Case group: sections of uterine leiomyoma tissue showing dense cellular content with diffuse collagen units (tailed arrow).

When the sections of uterine tissue from the control group stained with H&E and Masson’s trichrome stains were examined under a light microscope, normal smooth muscle cells were observed in the myometrium (Figure 1A,B and Figure 2A,B). In contrast, dense collagen deposits were observed between smooth muscle cells with dense cellular content in the sections of the uterine leiomyoma tissues in the case group (Figure 1C,D and Figure 2C,D). In line with this, fibroid sections were stained strongly with Masson’s trichrome, whereas myometrial sections showed weaker staining. Leiomyoma sections showed strong staining for STAT-3 and IL-26 compared with that in the normal myometrium.

### 3.2. Immunohistochemical Findings

#### 3.2.1. STAT-3 Primary Antibody

**Figure 3:**
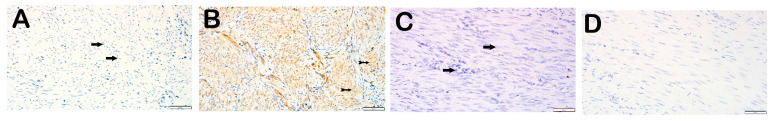
Representative light microscopic view of sections of biopsy specimens of uterine tissue incubated with STAT-3. (**A**) (×20) Control group: myometrium sections show that smooth muscle cells are negative for STAT-3 (arrow). Case Group (**B**) (×40): in leiomyoma tissue, an increased number of cells showing strong STAT-3 staining were observed in smooth muscle cells and fibroblasts (tailed arrow). Negative control group (**C**) (×40): myometrium sections show that smooth muscle cells are negative for STAT-3. Isotype control group (**D**) (×40): myometrium sections show that smooth muscle cells are negative for TRKB.

When uterine tissue sections of the control group incubated with STAT-3 primary antibody were examined under a light microscope, smooth muscle and fibroblast cells in the myometrium tissue were STAT-3-negative (Figure 3A, Table 2). In contrast, we observed an increased number of smooth muscle and fibroblast cells showing strong STAT-3 positivity in the uterine leiomyoma sections (Figure 3B, Table 2). As shown in Table 2, patients in the leiomyoma group had significantly higher STAT-3 h-scores than those in the control group (7.58 ± 2.27 vs. 0.25 ± 1.07, *p* < 0.001).

#### 3.2.2. IL-26 Primary Antibody

**Figure 4:**
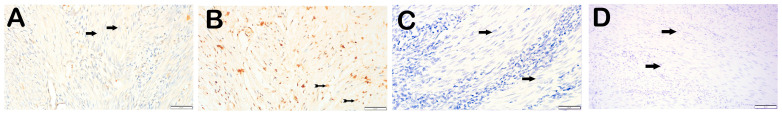
Representative light microscopic view of uterine tissue sections incubated with IL-26. (**A**) (×20) Control group: in myometrium sections, normal smooth muscle and fibroblast cells were negative for IL-26 (arrows). Case group (**B**) (×40): increased numbers of smooth muscle cells and fibroblast cells showing strong IL-26 staining in leiomyoma (tailed arrow). Negative control group (**C**) (×40): myometrium sections show that smooth muscle cells are negative for STAT-3. Isotype control group (**D**) (×40): myometrium sections show that smooth muscle cells are negative for TRKB.

When uterine tissue sections incubated with IL-26 primary antibody were examined under a light microscope, we found that normal smooth muscle and fibroblast cells in the control group were IL-26-negative (Figure 4A, Table 2). In contrast, we observed an increase in the number of cells showing high IL-26 positivity in smooth muscle cells and fibroblasts forming leiomyomas in the uterine tissue in the case group (Figure 4B, Table 2). In accordance with the light microscopic evaluation, patients in the leiomyoma group had significantly higher IL-26 h-scores than those in the control group (3.46 ± 2.21vs. 0.25 ± 0.61, *p* < 0.001).

The presence of STAT-3 and IL-26 in the myometrium and leiomyoma is shown in Figure 3 and Figure 4. The negative controls used to evaluate nonspecific staining are shown in Figure 3 and Figure 4. The h-score values for STAT-3 and IL-26 calculated for the fibroids and myometrium are shown in Table 2

## 4. Discussion

In this study, we investigated the expression levels of STAT-3 in uterine leiomyomas and evaluated the potential role of this transcription factor in fibroid pathogenesis. These findings suggest that STAT-3 is significantly increased in uterine leiomyomas and that this increase may be associated with various cellular mechanisms that support tumor growth.

Uterine leiomyomas are the most common benign tumors in women of reproductive age, and multiple mechanisms are involved in their development, including hormonal, genetic, and signaling pathway involvement. Recent studies have revealed that the STAT-3 pathway plays an important role in fibroid development.

STAT-3 is a transcription factor that is activated by cytokines and growth factors. After phosphorylation, it translocates to the nucleus and regulates several processes, including cell proliferation, the suppression of apoptosis, angiogenesis, and inflammatory response. These roles of STAT-3 make it an important regulator of tumorigenesis.

In a study conducted by Reschke et al. [13], it was demonstrated that the leptin hormone increases cell proliferation and extracellular matrix (ECM) accumulation in uterine leiomyoma cells through the JAK2/STAT-3 and MAPK/ERK pathways. Leptin exerts these effects by stimulating the phosphorylation of STAT-3 in leiomyoma cells. The same study also emphasized that leptin inhibitors suppress these processes [13].

Similarly, proinflammatory cytokines, such as interleukin-6 (IL-6), have been found to induce STAT-3 and increase the expression of ECM proteins (collagen I and fibronectin). Chegini reported that IL-6 increases proliferation and fibrotic response in leiomyoma cells via STAT-3 [14].

In addition, the microRNA-29 family (specifically, miR-29b) inhibits leiomyoma cell proliferation and migration by suppressing the STAT-3 signaling pathway. Huang et al. demonstrated that miR-29b inhibits the expression of proliferative genes, such as STAT-3, Cyclin D1, and c-Myc, thereby suppressing tumor growth [5].

These findings suggest that STAT-3 plays a multifaceted role in the growth and progression of uterine fibroids. STAT-3 regulates processes associated with tumor development, such as cell cycle progression, the inhibition of apoptosis, and ECM remodeling. Therefore, pharmacological targeting of STAT-3 early components in these signaling pathways may be a potential therapeutic strategy for controlling uterine fibroid growth. In particular, STAT-3 inhibitors are thought to be effective for hormone-independent treatment of fibroids.

Huang et al. [5]. reported that the miR-29 and the positivity of STAT-3 early components in these signaling pathways opened avenues for further investigation into their roles in other tumor types. Understanding these mechanisms may lead to broader applications in cancer treatment beyond uterine leiomyoma and benefit a wider patient population. These studies, with findings similar to ours, provide valuable insights that may lead to innovative treatment options, improve patient care, and guide future research in the field of gynecological tumors.

However, most existing studies in this field are based on cell cultures and animal models. Further translational and clinical research on human tissues is needed to elucidate the role of STAT-3 in the pathogenesis of uterine fibroids. Simultaneously, the clinical efficacy and safety of STAT-3 inhibitors should be evaluated in future studies.

Uterine leiomyomas (fibroids) are among the most common benign tumors in women, and their development involves various molecular pathways. One of these mechanisms involves interleukins (ILs) and other cytokines. However, there is limited information on the expression and effects of IL-26 in uterine fibroids in the existing literature.

Proinflammatory cytokines play an important role in the pathogenesis of uterine myoma. For example, changes in cytokine levels, such as IL-1β, IL-6, TNF-α, IL-8, IL-12p70, and IFN-γ have been observed. Konenkov et al. observed a significant decrease in IFN-γ levels and a tendency to decrease IL-1β and TNF-α levels in the sera of patients with uterine fibroids. These changes may adversely affect the proliferation and differentiation of the uterine tissues [15].

Furthermore, in another study, Isanbaeva et al. observed an increase in IL-1β, TGF-β2, and MCP-1 levels and a decrease in IL-2 levels in the serum of women with uterine fibroids. Changes in cytokine levels may be related to the growth of fibroids and may play a role in their progression [16].

Konenkov et al. [17] emphasized that the concentrations of important growth factors (IL-5, IL-7, G-CSF, VEGF, and PDGF) were decreased in the blood serum of women with uterine fibroids compared with healthy controls. Similarly, increased levels of peritoneal fluid interleukin-1 and the tumor necrosis factor in benign gynecological pathologies are evidence that uterine pathologies also affect the peritoneal microenvironment [18]. In line with this, in uterine fibroids, the phagocytic ability of neutrophils is affected by the serum levels of IL-2, IL-17A, IL-6, and IL-4. In endometrial cancer, the degranulation ability of neutrophils is affected by serum IL-18 levels. This finding may provide evidence that neutrophil behavior in the presence of uterine fibroids differs from that in endometrial cancer [19]. When these findings and our results are evaluated together, we suggest that fibroids affect the levels of different IL types in tissues, peritoneal fluid, and serum. The results may help healthcare providers understand the effects of uterine fibroids on fertility and reproductive outcomes. By understanding this role of growth, clinicians can better understand the characteristics and potential treatment options for this disease.

Since interleukins, along with other cytokines, trigger fibroid growth, IL-26 may also contribute to fibroid growth [17,20]. This idea is supported by the improvement in endometrial levels of many cytokines after the surgical resection of fibroids [21]. However, the lack of specific studies on the expression and effects of IL-26 in uterine fibroids makes it difficult to clarify the role of this cytokine in fibroid pathogenesis. Considering the role of IL-26 in other inflammatory diseases, it may act on uterine fibroids via similar mechanisms. Therefore, prospective studies are needed to better understand the expression and effects of IL-26 in uterine fibroids.

This study evaluated the expression of STAT-3 and IL-26, two early components of the relevant signaling pathways in leiomyoma tissue in uterine fibroid development; some limitations should be emphasized. Assessing IL-26 and STAT-3 solely by immunohistochemistry prevented us from reaching a definitive conclusion. If mRNA and protein analyses had been performed in addition to histological analysis, we could have provided clearer data on the etiopathology of fibroids. Furthermore, because the cycle phases of the patients were unknown at the time of surgery, STAT-3 and IL-26 expression may have been affected by different phases. Since we did not subdivide the fibroid group into submucosal, intramural, and subserosal groups, we cannot comment on the effects of myoma number and location on the expression of these markers. To confirm the significance of our findings in fibroid development, prospective studies with larger groups of participants and investigations of multiple markers at the histological, protein, and mRNA levels are needed, especially considering the heterogeneous nature of uterine leiomyomas. In addition, Upon re-evaluation of our experimental design and antibody selection, we acknowledge that the anti-STAT-3 antibody used in our immunohistochemical analysis recognizes total STAT-3 and is not phospho-specific. Therefore, our study does not fully address the functional activation of the STAT-3 signaling pathway, which depends on the phosphorylated (active) form of STAT-3. Future studies incorporating phospho-specific STAT-3 antibodies will be necessary to better assess the relationship between total and activated STAT-3 and to validate our current findings. In addition, the STAT-3 and IL-26 positivity in our study needs to be supported by studies evaluating the biomarkers of mononuclear cells (such as CD45, CD68, CD4, etc.) in order to distinguish them from myocytes and mononuclear cells.

## 5. Conclusions

In this study, we examined the levels of STAT-3 and IL-26 expression in samples obtained from patients with leiomyoma uteri and patients who underwent hysterectomy for benign reasons and evaluated the possible role of this transcription factor in the pathogenesis of uterine fibroids. The findings indicate that STAT-3 and IL-26 are significantly increased in uterine leiomyoma tissue, and that this increase may be associated with various cellular mechanisms that promote tumor growth. As most existing studies in this field are based on cell cultures and animal models, we believe that our study on human tissues will contribute significantly to the literature. While our study does not demonstrate that increased STAT-3 and IL-26 expressions are the sole determinant of fibroid development, it supports the involvement of signaling pathways associated with these two molecules in fibroid formation. In addition to their roles in tumorigenesis and angiogenesis, the fact that both cytokines activate inflammatory pathways suggests that multiple mechanisms are involved in the development of fibroids. However, additional experiments are needed to determine whether the upregulation of either of these markers has any impact on fibroid development or growth, particularly their ability to trigger inflammation and angiogenesis, which may make inhibiting these signaling pathways a therapeutic option for preventing fibroid development or shrinking existing fibroids. In future studies, pharmacologically targeting cytokine signaling pathways, such as STAT-3 or IL-26, may offer a potential treatment option to control the growth of uterine fibroids, which are very common in women of reproductive age.

## Figures and Tables

**Table 1:** Calculation of immunolabeling intensity according to the h-score formula.

Width	Corresponding Percentage Value	Staining Density	Corresponding Intensity
0	≤5%	0	No staining
0.1	≤25%	+0.5	Weak staining
0.4	>25–50%	+1	Low (×1)
0.6	51–75%	+2	Moderate staining (×2)
0.9	76–100%	+3	Strong staining (×3)

Histo-score = width × intensity.

**Table 2:** H-scores of the fibroid and control groups.

	Fibroid Group	Control Group	*p*-Value
STAT-3	7.58 ± 2.27	0.25 ± 1.07	<0.001
IL-26	3.46 ± 2.21	0.25 ± 0.61	<0.001

The results are expressed as mean ± standard error of the mean (SEM). An independent samples *t*-test was used to evaluate statistical significance.

## Data Availability

The corresponding author can provide the datasets generated and/or analyzed during this study upon reasonable request.
